# Cell Catcher: A New Method to Extract and Preserve Live Renal Cells from Urine

**DOI:** 10.34067/KID.0000000000000503

**Published:** 2024-09-26

**Authors:** Katia Nazmutdinova, Cheuk Yan Man, Martyn Carter, Philip L. Beales, Paul J.D. Winyard, Stephen B. Walsh, Karen L. Price, David A. Long

**Affiliations:** 1 Developmental Biology and Cancer Research and Teaching Department, University College London Great Ormond Street Institute of Child Health, London, United Kingdom; 2 University College London Centre for Kidney and Bladder Health, London, United Kingdom; 3 Encelo Laboratories Ltd., Harrow, United Kingdom; 4 B-made, The Bartlett School of Architecture, University College London, London, United Kingdom; 5 Genetics and Genomic Medicine Research and Teaching Department, University College London Great Ormond Street Institute of Child Health, London, United Kingdom; 6 Department of Renal Medicine, London Tubular Centre, University College London Medical School, London, United Kingdom

**Keywords:** cell biology and structure, epithelial, kidney, kidney disease, kidney tubule, podocyte, renal cell biology, renal epithelial cell, renal proximal tubule cell, renal tubular epithelial cells

## Introduction

A proportion of urinary tract cells are shed into urine by using normal physiological processes. These include epithelial cells from kidney tubules, podocytes, as well as immune and bladder cells.^1^ Urine-derived cells offer advantages over biopsies because they are easily obtained repeatedly without pain or discomfort. Furthermore, they have various research uses, including modeling genetic kidney disorders,^2^ generating stem cells,^3^ and drug screening.^4,5^ Despite these advantages, the full potential of urine-derived cells is not being realized.

One key issue is inconsistencies between methods to initiate cell cultures from urine. Healthy adult urine contains between 2.5 and 7.5 cells/100 ml, which can proliferate in culture, yielding millions of cells within 2–4 weeks.^6,7^ However, the success rate of initiating and expanding cells from urine is variable, ranging from 10% to 73%.^3,8–10^ In addition, the cell population obtained is heterogeneous, containing both differentiated and undifferentiated cells,^11^ complicating the interpretation of studies.

These inconsistencies in yields and cell identity are likely due to methodological differences, including culture conditions. Currently, urinary cells are isolated within 4 hours of sample collection, using two-step centrifugation, requiring a laboratory in close proximity to the collection site. This makes the process logistically challenging and extends the time cells are exposed to urine affecting cell viability.^1^

We hypothesized that immediate processing through filtration will improve the yield of cultured cells obtained from urine compared with centrifugation. This is because filtration minimizes urine exposure time, decreases processing times, and reduces mechanical stress on cells. To test this, we developed a filtration-based Cell Catcher device for processing urine at clinical sites and directly compared its efficiency with centrifugation.

## Methods

**Figure 1:**
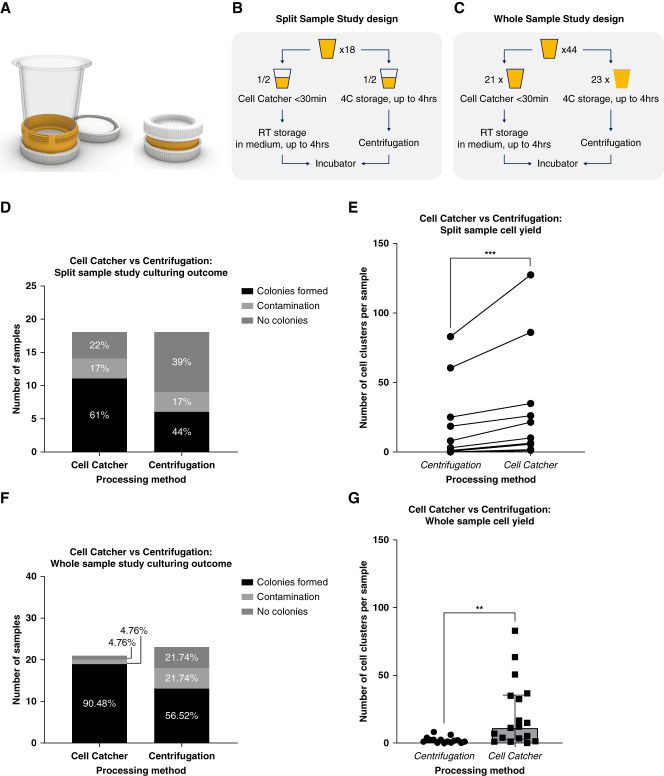
**Cell Catcher clinical validation study.** (A) Cell Catcher diagram. The hub of the Cell Catcher has detachable lids and houses a membrane. It connects to a detachable funnel for urine samples to be processed by gravity. After filtration, media are added for the cells to be preserved during transport, inside the hub. Prototypes were produced using Polyjet 3D printing (University College London, B-made 3D printing, Bartlett School of Architecture). (B) Split-sample study design. Eighteen samples were collected from patients with renal tubulopathies; each sample was split into two equal parts—Cell Catcher group and centrifugation group. Sample fractions in the Cell Catcher group were processed on site within 30 minutes of collection and stored at room temperature for up to 4 hours during transportation to the laboratory, where they were plated. Sample fractions in the centrifugation group were stored at 4°C and transported to the laboratory on ice within 4 hours to be centrifuged and plated. (C) Whole-sample study design. Forty-four samples were collected from patients with renal tubulopathies, BBS, and healthy adults, and whole volume was processed either by Cell Catcher or centrifugation. (D) Culturing outcomes, split-sample study. Cells from sample fractions processed by either a Cell Catcher or centrifugation were seeded, cultured, and assigned to the following categories: no clusters (by 2 weeks), clusters (day 6), and contamination (within the first day). Distribution of the three culturing outcomes for each experimental condition is shown. (E) Split-sample cell yield differences between Cell Catcher and centrifugation fractions. Cell clusters (>10 cells) were quantified on day 6 after plating, by two researchers independently. Average numbers of the two counts are plotted for each of the 11 samples in the paired study, where clusters formed in at least one of the fractions (Wilcoxon nonparametric paired *t* test, *n*=11, *P* = 0.001). (F) Culturing outcomes, whole-sample study. Cells from samples processed by either the Cell Catcher or centrifugation were seeded, cultured, and assigned to the following categories: no clusters (by 2 weeks), clusters (day 6), and contamination (within the first day). Distribution of the three culturing outcomes for each experimental condition is shown. (G) Whole-sample cell yield differences between Cell Catcher and centrifugation-processed samples. Cluster counts in samples processed by either Cell Catcher (*n*=18) or centrifugation (*n*=16), after two outliers that were identified in each group were removed. Mann–Whitney test, *P* = 0.0013. Median+interquartile range is plotted. BBS, Bardet–Biedl syndrome.

Urine was collected from consented patients at the Royal Free Hospital, St Thomas Hospital, and Great Ormond Street Hospital, London (Ethical approval references: 05/Q0508/6, 08/H0713/82). First, samples from 18 tubulopathy patients (Supplemental Table 1) were equally split by volume for paired analyses. One was processed by the Cell Catcher device (Figure 1A) within 30 minutes of collection at the clinic and the other transported on ice to a laboratory and centrifuged within 4 hours (Figure 1B). In a second study, we assessed samples from tubulopathy patients (*n*=18), adult and pediatric patients with Bardet–Biedl syndrome (BBS, *n*=15), and healthy controls (*n*=11) (Supplemental Table 2). In these 44 individuals, the whole sample volume was processed either by Cell Catcher or centrifugation (Figure 1C). Finally, a further six samples from tubulopathy patients were split and processed by either the Cell Catcher or centrifugation (Supplemental Table 3), and cell viability and phenotype were assessed before culture.

Cell Catcher is a patent-pending (Encelo Laboratories Ltd.) custom-built device manufactured using 3D printing that houses a polyethersulfone 5-*µ*m membrane (Sterlitech). Gravity-fed filtration can be achieved for samples <100 ml with low specific gravity (SG, 1.005–1.015) or <25 ml of high-SG samples (1.020–1.030) with all steps performed at room temperature. After filtration, the outer funnel was removed, a bottom lid attached to the hub, and 12 ml of medium (DMEM High Glucose/F12 [1:1], 1% penicillin/streptomycin, 1% amphotericin B, 10% FBS, human EGF, insulin, hydrocortisone, transferrin, triiodothyronine, epinephrine, bovine pituitary extract, and adenine) added to the cells contained in the upper portion of the hub. Centrifuged samples were processed as per published protocols.^12^ In samples assessed for viability before culture, cell pellets were resuspended in 10 *μ*l of medium, stained using trypan blue and counted by an automated machine (BioRad) to measure total cell count and percentage of live cells/ml of urine collected. The cells in three of these samples processed by the Cell Catcher were used for flow cytometry analysis. Cell pellets were resuspended in block (PBS with 5% mouse and rat serum) and incubated with allophycocyanin anti-human CD13 (Biolegend) and phycoerythrin anti-human podoplanin (Biolegend) antibodies as panepithelial and podocyte markers, respectively. Cells were washed, resuspended in PBS containing 2% FBS, and analyzed on FACSymphony A5 (BD Biosciences) with the population gated to exclude debris and dead cells.

All other cell samples were plated in a 12-well tissue culture dish and incubated at 37°C and 5% CO_2_. Half of the media was replaced with fresh once daily until day 3 and replaced completely every 2 days thereafter. Cells were passaged at around 80% confluence. Cultures were monitored daily and assigned to the following categories: contaminated (defined as bacterial infection within 1 day of culture, despite an initial negative result on urinalysis strips), formed cell clusters, and no clusters. Where cell clusters (>10 cells) formed, the number was counted on day 6 of culture independently by two investigators. RNA was extracted at first passage of the cells using the RNeasy Plus Mini kit (Qiagen); 500 ng was used to synthesize cDNA using the iScript gDNA Clear cDNA Synthesis Kit (BioRad). Transcripts of renal and bladder cell markers^13^ (Wilms tumor 1 [*WT1*], podocin [*NPHS2*], uroplakin 3A [*UPK3A*], uromodulin [*UMOD*], aquaporin-3 [*AQP3*], and aminopeptidase-A [*ENPEP*]) were assessed using RT-PCR. Glyceraldehyde-3-phosphate dehydrogenase (*GAPDH*) was used as a loading control, with RNA extracted from total kidney and bladder as positive controls. Primer details are available on request.

Data normality was assessed by Shapiro–Wilk test and significance assessed by *t* tests or Wilcoxon signed-rank nonparametric test. Data are presented as means±SD. Statistical significance was accepted at *P* < 0.05.

## Results

In our split-sample paired comparison, 61% of Cell Catcher samples formed cell clusters by day 6 compared with 44% of samples processed by centrifugation. Three samples were contaminated in each group (Figure 1D). In the 11 cultures that formed cell clusters in the Cell Catcher fraction, we counted the number and compared this directly in a paired analysis with the centrifugation samples from the same patients. The Cell Catcher–processed fractions contained significantly (*P* = 0.001) greater number of cell clusters (Figure 1E). In eight samples where cell clusters formed in both fractions, the number was on average double in the Cell Catcher–processed fraction (96% increase).

Similar observations were found when whole samples were processed using either Cell Catcher or centrifugation (Figure 1F). Ninety percent of Cell Catcher–processed samples (*n*=21) contained cell clusters, compared with 57% samples processed by centrifugation (*n*=23). The Cell Catcher–processed samples contained significantly higher number of clusters (11.5 versus 2, *P* = 0.0012, Figure 1G). There were no differences between the average sample volume (73.5 versus 74 ml) and patient age (26.3 versus 28.8 years) in the Cell Catcher and centrifugation groups. The centrifugation group had elevated average SG (1.014 versus 1.021, *P* < 0.05) and contained a greater proportion of female patients (43% versus 56%) and patients with BBS (29% versus 39%).

**Figure 2:**
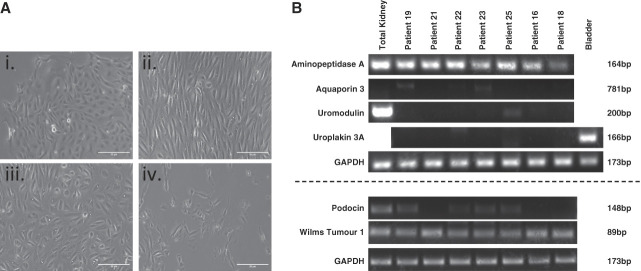
**Urine-derived cell characterization study results.** (A) Representative images of different cell morphologies observed in urine-derived cells. Variation in cell morphology was observed, with multiple types sometime present within 1 sample. Most commonly encountered proliferating types are (i–iii) with (iv) never reaching confluency. Cells with rice-grain morphology (iii) exhibited the best proliferative capacity, eventually becoming elongated (ii) and uniform with repeat passaging. Scale bar=50 *µ*m. (B) RT-PCR result summary. Tubulopathy patient-derived urinary cells were characterized using RT-PCR to detect transcripts of *ENPEP* (aminopeptidase A), *AQP3* (aquaporin 3), *UMOD* (uromodulin), *UPK3A* (uroplakin 3A), *NPHS2* (podocin), and *WT1* (Wilms tumor 1). *ENPEP* and *WT1* transcripts were detected in all patient samples.

Cells isolated using Cell Catcher were successfully expanded with yields of 0.5–2.2 million cells within 2 weeks. Examination by light microscopy revealed several cell morphologies (Figure 2A). From RNA extracted from seven tubulopathy patient cells, we detected *WT1* and *ENPEP* indicating podocyte and proximal tubule cells (Figure 2B). Some samples were also positive for *NPHS2*, but not *AQP3*, *UMOD*, or *UPK3A.* Cell cultures became phenotypically homogeneous with time, before ceasing to proliferate by weeks 4–6.

Before culture, we found similar numbers of cells captured in samples processed by Cell Catcher or centrifugation (8.2±5.4×10^5^ versus 8.9±3.7×10^5^ cells/ml of urine). A higher percentage of live cells were found in five of six samples processed by Cell Catcher (Supplemental Table 3), but no significant difference was found in average values compared with centrifugation (7.84±1.78 versus 6.26%±1.41%, *P* = 0.1). Flow cytometry analysis of three tubulopathy samples processed by the Cell Catcher found 82.1%±6.7% of cells before culture expressed CD13, with 35.2%±6.7% podoplanin positive.

## Discussion

We present a Cell Catcher device designed to standardize urine processing methods, to reduce the need for urgent laboratory processing, and to increase cell yield. In a paired analysis, we report a 17% increase in samples with viable cultured cells using Cell Catcher rather than centrifugation, with a two-fold increase of cells capable of attachment and proliferation. The Cell Catcher achieved a 90% cell cluster formation success rate when processing whole-volume, lower SG samples. The overall contamination rate was 14.5% (9/62) and seen predominantly in female patients (8/9), especially those with BBS. By defining optimal physical and biochemical urine properties (*e.g*., SG, volume) and improving collection protocols for easy mid-stream sampling, higher success rates could be realized. We found no difference between the cell number and viability obtained by Cell Catcher and centrifugation before culture. An explanation is that centrifugation may compromise the ability of the cells to attach and proliferate because of prolonged urine exposure. This would result in lower numbers of viable cultured cells but would not be detected by the trypan blue assay.

Urine-derived cells are well suited to study inherited kidney disease because a high number of physiologically relevant cells can be detected in patients with or being predisposed to renal dysfunctions caused by genetic conditions (BBS, Bartter syndrome, Dent disease). Similarly, higher yields have been observed in patients with other renal conditions (nephrotic syndrome, kidney stones, and Fanconi syndrome^8,10,14,15^). We detected transcripts of proximal tubule cell markers in all the cells we examined, with podocyte markers in some, confirming heterogeneity of cell populations shed in urine. Further research is needed to explore the device's compatibility with other cell types, including refinement of culturing conditions and medium formulations to isolate and/or expand other cell types known to be present in urine. In addition, the current Cell Catcher version's functionality is dependent on a sample's SG: iI works best with low-SG samples, which usually correlates with a high hydration level of the donor.

The Cell Catcher offers a distinct advantage over centrifugation by its potential compatibility with home use. By introducing the right preservation medium, the time frame for processing filtered samples can be extended, enabling direct shipment of live cells by patients. This innovation has the potential to advance methods of primary cell acquisition, by offering noninvasive, remote, and scalable procurement of live cells for clinical and research applications in the renal field and beyond.

## Supplementary Material

SUPPLEMENTARY MATERIAL

## Data Availability

The data collected for the study, including individual patient data and a data dictionary that defines each field in the dataset, will be made available as deidentified participant data to researchers who propose to use the data for individual patient data meta-analysis. Data will be shared following approval of the proposal by the corresponding author and a signed data access agreement.

## Supplemental Material

Supplemental Table 1. Split-sample study: a summary of donor profile, urine sample profile, and urine-derived cell culturing outcome.

Supplemental Table 2. Whole-sample study: a summary of donor profile, urine sample profile, and urine-derived cell culturing outcome.

Supplemental Table 3. Split-sample study: a summary of donor profile, urine sample profile, and urine-derived cell quantification before cell culture.
