# Supplementary material for: Cell Catcher: A New Method to Extract and Preserve Live Renal Cells from Urine
Source: Kidney360. 2024 Sep 26;5(9):1359–63. doi: 10.34067/KID.0000000000000503 (PMC11441801; doi:10.34067/KID.0000000000000503)
Supplement: SUPPLEMENTARY MATERIAL [file kidney360-5-1359-s001.pdf]

ASN American Society of Nephrology
JASN Journal of the American Society of Nephrology
KIDNEY360 Accessing Our World From Every Angle
CJASN Clinical Journal of the American Society of Nephrology

ASN Journal Disclosure Form

Date: 7/11/2024

Author: Philip Beales

Manuscript ID: K360-2024-000128R1

Manuscript Title: Cell Catcher: a new method to extract and preserve live renal cells from urine.

Disclosure Statements

Employer: University College London; Axovia Therapeutics
Ownership Interest: N/A
Consultancy: Rhythm Therapeutics LLC
Research Funding: N/A
Honoraria: N/A
Patents or Royalties: N/A
Advisory or Leadership Role: BBS-UK - SAB - Unpaid; Axovia Therapeutics - Board - Paid
Speakers Bureau: N/A
Other Interests or Relationships: N/A

ASN Journal Disclosure Form

As per ASN journal policy, I have disclosed any financial relationships or commitments I have held in the past 36 months as included below. I have listed my Current Employer below to indicate there is a relationship requiring disclosure. If no relationship exists, my Current Employer is not listed.

M. Carter reports the following:
Consultancy: Encelo Laboratories Limited

I understand that the information above will be published within the journal article, if accepted, and that failure to comply and/or to accurately and completely report the potential financial conflicts of interest could lead to the following: 1) Prior to publication, article rejection, or 2) Post-publication, sanctions ranging from, but not limited to, issuing a correction, reporting the inaccurate information to the authors' institution, banning authors from submitting work to ASN journals for varying lengths of time, and/or retraction of the published work.

Name: Martyn E Carter
Manuscript ID: K360-2024-000128R1
Manuscript Title: Cell Catcher: a new method to extract and preserve live renal cells from urine.
Date of Completion: July 4, 2024
Disclosure Updated Date: July 4, 2024

ASN American Society of Nephrology
JASN Journal of the American Society of Nephrology
KIDNEY360 Accessing Our World From Every Angle
CJASN Clinical Journal of the American Society of Nephrology

ASN Journal Disclosure Form

As per ASN journal policy, I have disclosed any financial relationships or commitments I have held in the past 36 months as included below. I have listed my Current Employer below to indicate there is a relationship requiring disclosure. If no relationship exists, my Current Employer is not listed.

D. Long reports the following:
Employer: University College London; and Ownership Interest: Encelo Laboratories.

I understand that the information above will be published within the journal article, if accepted, and that failure to comply and/or to accurately and completely report the potential financial conflicts of interest could lead to the following: 1) Prior to publication, article rejection, or 2) Post-publication, sanctions ranging from, but not limited to, issuing a correction, reporting the inaccurate information to the authors' institution, banning authors from submitting work to ASN journals for varying lengths of time, and/or retraction of the published work.

Name: David A. Long
Manuscript ID: K360-2024-000128R1
Manuscript Title: Cell Catcher: a new method to extract and preserve live renal cells from urine
Date of Completion: July 2, 2024
Disclosure Updated Date: July 2, 2024

ASN American Society of Nephrology
JASN Journal of the American Society of Nephrology
KIDNEY360 Accessing Our World From Every Angle
CJASN Clinical Journal of the American Society of Nephrology

ASN Journal Disclosure Form

As per ASN journal policy, I have disclosed any financial relationships or commitments I have held in the past 36 months as included below. I have listed my Current Employer below to indicate there is a relationship requiring disclosure. If no relationship exists, my Current Employer is not listed.

C. Man reports the following:
Employer: ENCELO LABORATORIES LIMITED; Consultancy: ENCELO LABORATORIES LIMITED; Research Funding: ENCELO LABORATORIES LIMITED; and Patents or Royalties: ENCELO LABORATORIES LIMITED.

I understand that the information above will be published within the journal article, if accepted, and that failure to comply and/or to accurately and completely report the potential financial conflicts of interest could lead to the following: 1) Prior to publication, article rejection, or 2) Post-publication, sanctions ranging from, but not limited to, issuing a correction, reporting the inaccurate information to the authors' institution, banning authors from submitting work to ASN journals for varying lengths of time, and/or retraction of the published work.

Name: Cheuk Yan Man
Manuscript ID: K360-2024-000128R1
Manuscript Title: Cell Catcher: a new method to extract and preserve live renal cells from urine.
Date of Completion: July 2, 2024
Disclosure Updated Date: July 2, 2024

ASN
American Society of Nephrology
JASN
Journal of the American Society of Nephrology
KIDNEY360
Accessing Our World From Every Angle
CJASN
Clinical Journal of the American Society of Nephrology

ASN Journal Disclosure Form

As per ASN journal policy, I have disclosed any financial relationships or commitments I have held in the past 36 months as included below. I have listed my Current Employer below to indicate there is a relationship requiring disclosure. If no relationship exists, my Current Employer is not listed.

K. Nazmutdinova reports the following:
Employer: University College London; Ownership Interest: Encelo Laboratories Ltd; Research Funding: Encelo Laboratories Ltd; Patents or Royalties: Encelo Laboratories Ltd; and Advisory or Leadership Role: Encelo Laboratories Ltd.

I understand that the information above will be published within the journal article, if accepted, and that failure to comply and/or to accurately and completely report the potential financial conflicts of interest could lead to the following: 1) Prior to publication, article rejection, or 2) Post-publication, sanctions ranging from, but not limited to, issuing a correction, reporting the inaccurate information to the authors' institution, banning authors from submitting work to ASN journals for varying lengths of time, and/or retraction of the published work.

Name: Katia Nazmutdinova
Manuscript ID: K360-2024-000128R1
Manuscript Title: Cell Catcher: a new method to extract and preserve live renal cells from urine
Date of Completion: July 3, 2024
Disclosure Updated Date: July 3, 2024

ASN American Society of Nephrology | JASN Journal of the American Society of Nephrology | KIDNEY360 Accessing Our World From Every Angle | CJASN Clinical Journal of the American Society of Nephrology

ASN Journal Disclosure Form

As per ASN journal policy, I have disclosed any financial relationships or commitments I have held in the past 36 months as included below. I have listed my Current Employer below to indicate there is a relationship requiring disclosure. If no relationship exists, my Current Employer is not listed.

K. Price has nothing to disclose.

I understand that the information above will be published within the journal article, if accepted, and that failure to comply and/or to accurately and completely report the potential financial conflicts of interest could lead to the following: 1) Prior to publication, article rejection, or 2) Post-publication, sanctions ranging from, but not limited to, issuing a correction, reporting the inaccurate information to the authors' institution, banning authors from submitting work to ASN journals for varying lengths of time, and/or retraction of the published work.

Name: Karen Price
Manuscript ID: K360-2024-000128R1
Manuscript Title: Cell Catcher: a new method to extract and preserve live renal cells from urine
Date of Completion: June 24, 2024
Disclosure Updated Date: June 24, 2024

ASN Journal Disclosure Form

As per ASN journal policy, I have disclosed any financial relationships or commitments I have held in the past 36 months as included below. I have listed my Current Employer below to indicate there is a relationship requiring disclosure. If no relationship exists, my Current Employer is not listed.

S. Walsh reports the following:
Employer: UCL; and Consultancy: Advicienne.

I understand that the information above will be published within the journal article, if accepted, and that failure to comply and/or to accurately and completely report the potential financial conflicts of interest could lead to the following: 1) Prior to publication, article rejection, or 2) Post-publication, sanctions ranging from, but not limited to, issuing a correction, reporting the inaccurate information to the authors' institution, banning authors from submitting work to ASN journals for varying lengths of time, and/or retraction of the published work.

Name: Stephen B. Walsh
Manuscript ID: K360-2024-000128R1
Manuscript Title: Cell Catcher: a new method to extract and preserve live renal cells from urine
Date of Completion: July 3, 2024
Disclosure Updated Date: July 3, 2024

ASN
American Society of Nephrology
JASN
Journal of the American Society of Nephrology
KIDNEY360
Accessing Our World From Every Angle
CJASN
Clinical Journal of the American Society of Nephrology

ASN Journal Disclosure Form

As per ASN journal policy, I have disclosed any financial relationships or commitments I have held in the past 36 months as included below. I have listed my Current Employer below to indicate there is a relationship requiring disclosure. If no relationship exists, my Current Employer is not listed.

P. Winyard has nothing to disclose.

I understand that the information above will be published within the journal article, if accepted, and that failure to comply and/or to accurately and completely report the potential financial conflicts of interest could lead to the following: 1) Prior to publication, article rejection, or 2) Post-publication, sanctions ranging from, but not limited to, issuing a correction, reporting the inaccurate information to the authors' institution, banning authors from submitting work to ASN journals for varying lengths of time, and/or retraction of the published work.

Name: Paul Winyard
Manuscript ID: K360-2024-000128R1
Manuscript Title: Cell Catcher: a new method to extract and preserve live renal cells from urine
Date of Completion: June 26, 2024
Disclosure Updated Date: June 26, 2024
