# Supplementary material for: Cell Catcher: A New Method to Extract and Preserve Live Renal Cells from Urine
Source: Kidney360. 2024 Sep 26;5(9):1359–63. doi: 10.34067/KID.0000000000000503 (PMC11441801; doi:10.34067/KID.0000000000000503)
Supplement: SUPPLEMENTARY MATERIAL [file kidney360-5-1359-s002.pdf]

Supplementary Data

Cell Catcher: a new method to extract and preserve live renal cells from urine.

Katia Nazmutdinova, Cheuk Yan Man, Martyn Carter, Philip L Beales, Paul JD Winyard, Stephen B Walsh, Karen L Price, David A Long

Table of Contents

Supplementary Table 1. Split sample study: a summary of donor profile, urine sample profile and urine-derived cell culturing outcome.

Supplementary Table 2. Whole sample study: a summary of donor profile, urine sample profile and urine-derived cell culturing outcome.

Supplementary Table 3. Split sample study: a summary of donor profile, urine sample profile and urine-derived cell quantification prior to cell culture.

Supplementary Table 1. Split sample study: a summary of donor profile, urine sample profile and urine-derived cell culturing outcome. Number of colonies per sample is the average of the counts by two investigators on Day 6 of cell culture. *SG – Specific Gravity, Cont. – contamination, BBS – Bardet-Biedl Syndrome*

	**Donor profile**			**Urine sample profile**		**Cell culturing outcome**	
**Sample ID**	Age group	Gender	Clinical diagnosis	Volume, ml	SG	Processed by Cell Catcher, 1/2 of the total volume, (number of clusters)	Processed by Centrifugation, 1/2 of the total volume, (number of clusters)
**1**	50-69	M	Hypokalaemic Hypertension	85	1.020	Cont.	Cont.
**2**	30-49	F	Medullary Nephrocalcinosis	50	1.030	(0)	(0)
**3**	70-89	F	Tubulointerstitial Nephritis	30	1.020	Cont.	Cont.
**4**	50-69	F	IgA nephropathy	80	1.020	(21.5)	(8)
**5**	50-69	M	Tubulointerstitial Nephritis	30	1.025	(5.5)	(0)
**6**	18-29	F	Distal renal tubular acidosis (dRTA)	40	1.010	(35)	(25)
**7**	18-29	M	Distal renal tubular acidosis (dRTA)	50	1.015	(26)	(18.5)
**8**	30-49	M	Kidney stones	28	1.015	(0)	(0)
**9**	50-69	F	Distal Salt Losing Tubulopathy	38	1.020	(0)	(0)
**10**	30-49	F	Nephrogenic Diabetes Insipidus	75	1.005	(0)	(0)
**11**	50-69	F	Tubulointerstitial Nephritis	20	1.010	(2)	(0)
**12**	30-49	M	Distal salt losing tubulopathy	40	1.025	(1)	(0)
**13**	50-69	F	Tubulointerstitial Nephritis	80	1.025	(10)	(3)
**14**	30-49	F	Not yet diagnosed	100	1.015	(1.5)	(1)
**15**	30-49	F	Distal salt losing tubulopathy	100	1.015	Cont.	Cont.
**16**	18-29	M	Proximal Tubulopathy	100	1.020	(86)	(60.5)
**17**	50-69	M	Distal Tubulopathy	60	1.020	(6.5)	(1)
**18**	18-29	M	Proximal tubulopathy	60	1.025	(127.5)	(83)

Supplementary Table 2. Whole sample study: a summary of donor profile, urine sample profile and urine-derived cell culturing outcome. Number of colonies per sample is the average of the counts by two investigators on Day 6 of cell culture. *SG – Specific Gravity, CC- Cell Catcher. CF – Centrifugation, BBS – Bardet-Biedl Syndrome, CKD – Chronic Kidney Disease, Cont. - contamination, NR – not recorded*

Sample ID	Age range	Gender	Clinical diagnosis	Sample Volume, ml	Sample SG	Processing method	Cell culturing outcome (number of clusters)
**19**	18-29	F	Pseudo-Bartter syndrome	55	1.010	CC	(35)
**20**	50-69	M	Renal vascular disease / CKD3	17	1.030	CC	(0)
**21**	30-49	M	Hyperphosphataemia	80	1.010	CC	(32.5)
**22**	30-49	M	Distal Salt losing Tubulopathy	130	1.020	CC	(36.5)
**23**	18-29	F	Proximal Tubulopathy	120	1.020	CC	(50.5)
**24**	30-49	F	Proximal Tubulopathy	100	1.020	CC	(63.5)
**25**	18-29	M	Distal Salt Losing Tubulopathy	100	1.020	CC	(195.5)
**26**	30-49	F	Nephrocalcinosis	80	1.010	CC	(15)
**27**	30-49	F	Kidney stones	20	1.020	CC	(16.5)
**28**	18-29	M	Distal Salt Losing Tubulopathy	105	1.015	CC	(83)
**29**	18-29	M	BBS	35	1.020	CC	(121)
**30**	18-29	M	BBS	60	1.000	CC	(12)
**31**	4-10	M	BBS	60	1.000	CC	(1)
**32**	11-17	F	BBS	38	1.005	CC	(11)
**33**	11-17	F	BBS	78	1.005	CC	Cont.
**34**	4-10	F	BBS	50	1.025	CC	(4)
**35**	18-29	F	Control	100	1.010	CC	(7)
**36**	18-29	M	Control	95	1.015	CC	(5)
**37**	NR	NR	Control	NR	1.010	CC	(1.5)
**38**	NR	NR	Control	NR	1.015	CC	(3.5)
**39**	NR	NR	Control	NR	1.020	CC	(1)
**40**	50-69	F	Distal renal tubular acidosis (dRTA)	100	1.015	CF	Cont.
**41**	30-49	M	Hereditary Renal Phosphate disorder	100	1.020	CF	(0)
**42**	30-49	M	Hypertension	80	1.030	CF	(11.5)
**43**	30-49	M	Fanconi-Bickel syndrome	100	1.030	CF	(15.5)
**44**	18-29	M	Nephrogenic diabetes insipidus	95	1.005	CF	(0)
**45**	18-29	F	Distal Salt Losing tubulopathy	80	1.020	CF	(8)
**46**	18-29	M	Distal Salt Losing Tubulopathy	60	1.030	CF	(2.5)
**47**	18-29	F	Hereditary Renal Phosphate disorder	70	1.030	CF	(0)
**48**	30-49	F	BBS	30	NR	CF	(1)
**49**	30-49	F	BBS	100	1.025	CF	(34)
**50**	18-29	F	BBS	100	NR	CF	Cont.

**51**	11-17	F	BBS	50	1.015	CF	Cont.
**52**	11-17	F	BBS	53	NR	CF	(2.5)
**53**	11-17	F	BBS	18	NR	CF	Cont.
**54**	11-17	F	BBS	44	1.025	CF	(2)
**55**	4-10	F	BBS	100	1.025	CF	Cont.
**56**	11-17	F	BBS	50	1.030	CF	(6)
**57**	30-49	M	Control	60	1.025	CF	(1)
**58**	30-49	M	Control	100	1.010	CF	(2.5)
**59**	30-49	F	Control	100	1.015	CF	(0)
**60**	NR	NR	Control	50	1.020	CF	(1)
**61**	NR	NR	Control	85	1.020	CF	(0)
**62**	NR	NR	Control	80	1.015	CF	(4)

Supplementary Table 3. Split sample study: a summary of donor profile, urine sample profile and urine-derived cell quantification prior to cell culture. Proportion of live cells per 1ml of processed urine sample captured by each method, average of two counts is shown. *SG – Specific Gravity, NR – not recorded*

	**Donor profile**			**Urine sample profile**		**Cell quantification prior to cell culture**		
**Sample ID**	Age group	Gender	Clinical diagnosis	Volume, ml	SG	Processed by Cell Catcher, % of live cells, per 1ml of urine	Processed by Centrifugation, % of live cells, per 1ml of urine	Relative change in % of live cells, Cell Catcher versus Centrifugation
**63**	18-29	M	Bartter Syndrome	35	1.020	10.32%	5.88%	75.53
**64**	18-29	M	Distal renal tubular acidosis	37.5	1.015	5.81%	4.61%	26.06
**65**	18-29	M	Nephrogenic diabetes insipidus	130	1.005	7.19 %	4.87%	47.69
**66**	50-69	F	Distal renal tubular acidosis	82	1.010	10.28%	8.08%	27.23
**67**	40-49	M	Hypertension	84	NR	6.72%	5.99%	12.29
**68**	30-39	M	Nephrocalcinosis	108	NR	6.75%	8.18%	-17.42
